# Hierarchical graph transformer with contrastive learning for protein function prediction

**DOI:** 10.1093/bioinformatics/btad410

**Published:** 2023-06-27

**Authors:** Zhonghui Gu, Xiao Luo, Jiaxiao Chen, Minghua Deng, Luhua Lai

**Affiliations:** Peking-Tsinghua Center for Life Sciences, Academy for Advanced Interdisciplinary Studies, Peking University, Beijing 100871, China; Department of Computer Science, University of California, Los Angeles, Los Angeles, CA 90024, United States; Center for Quantitative Biology, Academy for Advanced Interdisciplinary Studies, Peking University, Beijing 100871, China; Center for Quantitative Biology, Academy for Advanced Interdisciplinary Studies, Peking University, Beijing 100871, China; School of Mathematics Sciences, Peking University, Beijing 100871, China; Peking-Tsinghua Center for Life Sciences, Academy for Advanced Interdisciplinary Studies, Peking University, Beijing 100871, China; Center for Quantitative Biology, Academy for Advanced Interdisciplinary Studies, Peking University, Beijing 100871, China; BNLMS, College of Chemistry and Molecular Engineering, Peking University, Beijing 100871, China

## Abstract

**Motivation:**

In recent years, high-throughput sequencing technologies have made large-scale protein sequences accessible. However, their functional annotations usually rely on low-throughput and pricey experimental studies. Computational prediction models offer a promising alternative to accelerate this process. Graph neural networks have shown significant progress in protein research, but capturing long-distance structural correlations and identifying key residues in protein graphs remains challenging.

**Results:**

In the present study, we propose a novel deep learning model named Hierarchical graph transformEr with contrAstive Learning (HEAL) for protein function prediction. The core feature of HEAL is its ability to capture structural semantics using a hierarchical graph Transformer, which introduces a range of super-nodes mimicking functional motifs to interact with nodes in the protein graph. These semantic-aware super-node embeddings are then aggregated with varying emphasis to produce a graph representation. To optimize the network, we utilized graph contrastive learning as a regularization technique to maximize the similarity between different views of the graph representation. Evaluation of the PDBch test set shows that HEAL-PDB, trained on fewer data, achieves comparable performance to the recent state-of-the-art methods, such as DeepFRI. Moreover, HEAL, with the added benefit of unresolved protein structures predicted by AlphaFold2, outperforms DeepFRI by a significant margin on Fmax, AUPR, and Smin metrics on PDBch test set. Additionally, when there are no experimentally resolved structures available for the proteins of interest, HEAL can still achieve better performance on AFch test set than DeepFRI and DeepGOPlus by taking advantage of AlphaFold2 predicted structures. Finally, HEAL is capable of finding functional sites through class activation mapping.

**Availability and implementation:**

Implementations of our HEAL can be found at https://github.com/ZhonghuiGu/HEAL.

## 1 Introduction

Recent development in high-throughput sequencing has resulted in a great increase in the number of protein sequences in benchmark databases such as (Apweiler *et al.* 2004, UniProt Consortium 2019). However, the bulk of protein sequences lack functional annotation owing to the exorbitant expense and low-throughput experimental studies (Radivojac *et al.* 2013, Zhou *et al.* 2019). Therefore, computational approaches that can automatically and precisely deduce protein functions are much wanted. Commonly used methods for inferring functions for a new protein sequence include sequence-alignment that identify similar domains (FunFam) (Das *et al.* 2015) or local alignments (Blast) (Altschul *et al.* 1990, Buchfink *et al.* 2015), to transfer the functions of proteins that have been experimentally confirmed before. With the advance of machine learning, a variety of computational approaches for protein function prediction have been developed (Yang *et al.* 2015, Fa *et al.* 2018, Kulmanov *et al.* 2018, Gelman *et al.* 2021). In the Critical Assessment of Functional Annotation (CAFA), a blind prediction challenge, machine learning methods have demonstrated superior performance compared to traditional sequence alignment-based methods (Radivojac *et al.* 2013). These machine learning methods can be broadly categorized into knowledge-based, sequence-based, and structure-based approaches. Knowledge-based approaches typically incorporate information from external sources such as protein–protein interaction (PPI) networks (Mostafavi *et al.* 2008, Cho *et al.* 2016, You *et al.* 2021). However, the absence of prior knowledge may limit their practical analysis of newly discovered protein sequences (Gligorijević *et al.* 2021). Sequence-based approaches often use primary sequence as well as some other hand-crafted features to predict protein functions (Fa *et al.* 2018, Kulmanov *et al.* 2018, Zhang *et al.* 2019, Cao and Shen 2021, Kulmanov and Hoehndorf 2021, Yao *et al.* 2021, Zhu *et al.* 2022). Additionally, since structural information has a direct connection with protein functions, structure-based methods have become increasingly popular (Gligorijević *et al.* 2021, Lai and Xu 2022, Zhao *et al.* 2022). These methods utilize both protein structural and sequential information for function prediction.

Recent advances in deep learning have led to the development of various effective techniques for protein function prediction. Sequence-based approaches (Fa *et al.* 2018, Kulmanov *et al.* 2018, Zhang *et al.* 2019, Cao and Shen 2021, Wang *et al.* 2023) relied solely on one-dimensional (1D) convolutional neural networks (CNNs) or Transformer models to create discriminative protein sequence representations. Later, methods combining both query sequence and homology information showed significant improvements (Kulmanov and Hoehndorf 2021, Zhu *et al.* 2022). Another approach involves integrating literature information with sequence information extracted by recurrent neural networks (Yao *et al.* 2021). As three-dimensional (3D) structures have a direct relationship with functions and structural homologs can have highly diverse sequences, relying solely on sequence-based methods can become a major bottleneck. With the recent development in protein structure prediction research, it has become easier to get protein contact maps or even 3D structures (Baek *et al.* 2021b, Jumper *et al.* 2021). Furthermore, deep learning techniques for structured data have seen significant improvements, leading to the emergence of structure-based methods that can fully utilize protein structural data through deep learning (Gligorijević *et al.* 2021, Lai and Xu 2022, Zhao *et al.* 2022). These methods typically model 3D structures using graphs and then employ the structural information using graph neural networks (GNNs) (Kipf and Welling 2016) following the message passing paradigm (Gilmer *et al.* 2020). Specifically, each residue receives signals from its geometric neighborhood, which are aggregated to update its representation at each layer. Finally, a graph pooling layer is used to summarize all the residue representations into a protein representation for downstream classification. Among structure-based methods, DeepFRI (Gligorijević *et al.* 2021) was the first to leverage protein structures built by homology modeling for reinforcement, achieving state-of-the-art performance with good interpretability. Subsequently, GAT-GO (Lai and Xu 2022) was the first to utilize contact maps predicted by a structure prediction neural network to learn protein functions.

In spite of recent advances in protein function prediction using GNN-based approaches, the following limitations remain to be solved: (i) Long-distance structural correlations are difficult to be included. Due to the major oversmoothing problem, existing methods usually adopt shallow GNNs. The restriction on network depths makes it hard to explore long-distance spatial patterns in 3D protein structures. (ii) It is hard to capture residues that are crucial for protein function. Protein representations are often generated using a simple graph pooling procedure that takes the average or maximum of all residue representations, treating all residues equally despite the fact that protein functionality often depends on specific residues. Therefore, an effective procedure needs to be envisaged to generate protein representations in an adaptive manner.

To tackle the aforementioned limitations, we propose a novel method, Hierarchical graph transformEr with contrAstive Learning (HEAL) for protein function prediction in this study. Our approach involves constructing a graph input based on both sequential features and the contact map, followed by the collection of short-distance information using message passing neural networks. We then introduce a hierarchical graph Transformer to explore long-distance correlations and aggregate the node representation in a self-adaptive manner. To learn topological semantics, we first introduce a set of super-nodes that interact with nodes in the protein graph. We then use the self-attention mechanism to aggregate the semantic super-node representations into a graph representation. Additionally, we incorporate graph contrastive learning (Kumar *et al.* 2022) by smoothly perturbing node features and increasing the similarity score between the representations of different views. This objective is applied as a regularized term to optimize the model along with supervised learning.

We conduct extensive experiments to compare HEAL with baseline methods including Blast (Altschul *et al.* 1990), FunFam (Das *et al.* 2015), DeepGO (Kulmanov *et al.* 2018), DeepGOPlus (Kulmanov and Hoehndorf 2021), and DeepFRI (Gligorijević *et al.* 2021), in various settings. To evaluate our proposed method, we first trained the model using proteins from the Protein Data Bank (PDB) (Berman *et al.* 2000), and the resulting model, HEAL-PDB, exhibited performance comparable to DeepFRI. We then enhanced the model by incorporating AlphaFold2 (AF2)-predicted protein structures, and HEAL outperforms DeepGO and DeepFRI on PDBch test set. On the test set of AF2 predicted structures (AFch test set), HEAL is very robust and performs better than other state-of-art methods, DeepGOPlus and DeepFRI. Our model also demonstrates outstanding generalizability and excellent interpretability, allowing for the identification of functionally significant residues.

## 2 Materials and methods

### 2.1 An overview of HEAL

**Figure 1.**
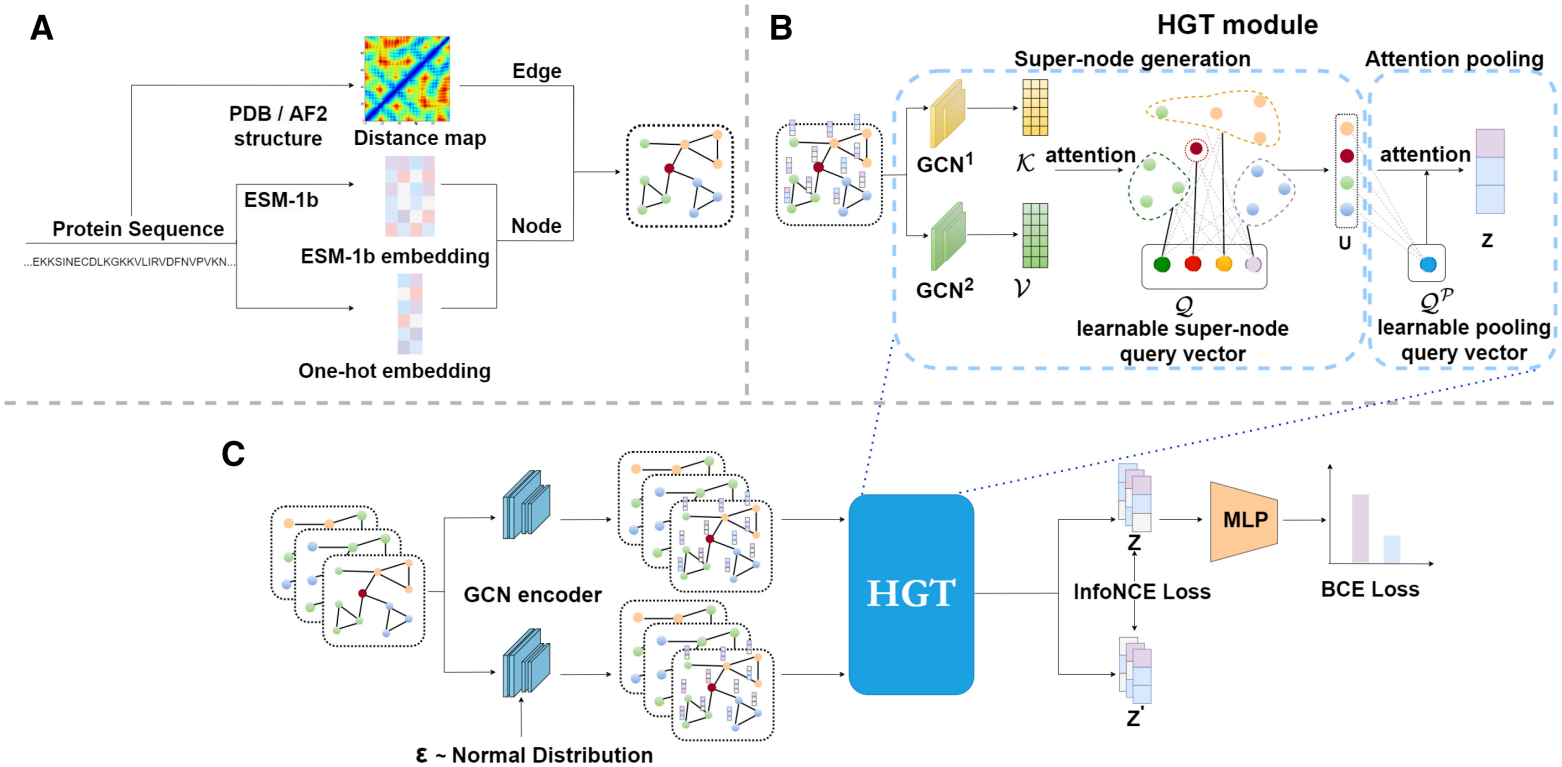
Overview of the proposed HEAL. (A) The flowchart for building a protein graph. The residue embeddings are derived from a combination of one-hot embeddings and ESM-1b language embeddings (Rives *et al.* 2021). The edge information is obtained from the distance map to connect the residues. (B) The overview of the hierarchical graph Transformer (HGT) module. We utilize GCN layers to get the topological key and value vectors of the graphs. Subsequently, following the attention paradigm, the learnable super-node query vectors interact with the key and value vectors, aggregating the original nodes into super-node representations (***U***). Similar to the super-node generation process described earlier, we use 1D learnable pooling query vectors to help pool the super-node representations into graph-level representations (***z***). (C) The overview of HEAL. We feed a batch of protein graphs into the GCN encoder, and the node embeddings are perturbed to provide a different view. These resulting node embeddings are then pooled into graph-level representations by the HGT module. The functions are predicted by an MLP, and the model is optimized using both binary cross-entropy loss for function classification and InfoNCE loss for different views of graph-level representations (***z*** and z′).

We first construct graph input for each protein. The main architecture of HEAL consists of two parts, message passing neural network and hierarchical graph Transformer. We then optimize the network using both supervised learning and contrastive learning. More information can be found in Fig. 1.

### 2.2 Graph input

To explore geometric information, we characterize each protein using a graph G=(V,E) where V and E are node and edge sets, respectively. We first extract feature embeddings and then infer the graph structure from its contact map (Fig. 1A).

**Feature Extractor.** In HEAL, node features is obtained from two aspects as follows: (i) One-hot residue encoder: each sequence is encoded by one of amino acid symbols. (ii) ESM-1b protein language model (Rives *et al.* 2021): a large-scale protein language model, which produces the residue embeddings to capture intrinsic protein knowledge. We concatenate the above embeddings $x_{v}\in R^{F}$ for each node *v*, producing an informative node feature matrix $X\in R^{L\times F}$ for each protein graph *G* with *L* residues.

**Structural Mining.** After extracting node features, we infer the structural information from the contact map. In detail, we first obtain 3D atomic coordinates of each protein from PDB (Berman *et al.* 2000). Then we add an edge between two nodes if the distance between their $C_{\alpha }$ atoms is less than 10 Å. In summary, the $C_{\alpha }-C_{\alpha }$ contact map serves as the adjacent matrix $A\in R^{L\times L}$ for each protein graph *G*.

### 2.3 Message passing neural network

To collect local information in the protein graph, we first adopt a message passing neural network (Fig. 1C GCN encoder), where neighborhood information of each node are aggregated for updating central node representations (Kipf and Welling 2016). In particular, let $H^{0}=X$ denote the initial hidden embedding matrix and we update the hidden embeddings as in: in which $\tilde{A}=A+I$ denotes the adjacent matrix with self-loops added and $\tilde{D}$ is the diagonal degree matrix for normalization. After *N* message passing layers, we generate the hidden embedding matrix $H\in R^{L\times D}$ where *D* denotes the hidden dimension by $H=H^{N}=\text{MPNN}(X,A)$ with sufficient local geometric information embedded.


(1) $$H^{n+1}=\text{ReLU}\left({\tilde{D}}^{-0.5}\tilde{A}{\tilde{D}}^{-0.5}H^{\left(n\right)}W^{\left(n\right)}\right)$$

### 2.4 Hierarchical graph transformer

Previous methods usually directly adopt a global pooling layer such as averaging or sum to summarize these node embeddings in ***H*** (Gligorijević *et al.* 2021, Lai and Xu 2022, Zhao *et al.* 2022). However, this strategy is incapable of recognizing important nodes. Even worse, they cannot infer long-distance structural relationships in protein graphs. To tackle this, motivated by recent Transformer models (Baek *et al.* 2021a), we introduce a hierarchical graph Transformer, which contains learnable super-nodes to explore long-distance correlations, followed by a attention module (Vaswani *et al.* 2017) to generate graph-level representations (Fig. 1B).

**Super-node Generation.** We introduce *K* super-nodes with learnable features, $q_{1},\ldots ,q_{K}$. These super-nodes are expected to interact with node embeddings in the specific protein graph for the exploration of global structural information. Motivated by the recent Transformer models (Vaswani *et al.* 2017), we regard super-nodes as query vectors, whereas the key and value vectors are both from hidden embeddings with additional message passing neural networks. In formulation, we calculate the similarity between each query vector and key vectors, which serves as weights to summarize all the value vectors. Concatenating all super-node query representations into $Q\in R^{K\times D}$, we can aggregate all the nodes into *K* super-node representations $\Gamma \in R^{K\times D}$ with topological semantics. In formulation, the updated super-node embedding matrix is: where ${\text{GCN}}^{1}$ and ${\text{GCN}}^{2}$ denote other encoders to further aggregate the hidden features to get the key matrix $K\in R^{L\times D}$ and value matrix $V\in R^{L\times D}$. To maximize the model capacity, we construct multihead super-node embedding matrices with distinct network parameters, i.e. $\Gamma_{1},\ldots ,\Gamma_{H}$. Then, we concatenate all these matrices and utilize a fully connected layer to generate semantics-aware super-node representations. In formulation, we generate semantics-aware super-node embedding matrix $U\in R^{K\times D}$ as: where $FC^{1}(\cdot )$ is a multilayer perceptron (MLP) to transform the embeddings for each super-node.


(2) $$\Gamma =\text{softmax}\left(\frac{Q\cdot K^{⊤}}{\sqrt{D}}\right)\cdot V,$$

(3) $$K={\text{GCN}}^{1}(H,A),\;V={\text{GCN}}^{2}(H,A)$$

(4) $$U=FC^{1}([\Gamma_{1},\ldots ,\Gamma_{H}])$$

**Attention Pooling.** Note that these semantics-aware super-node representations are obtained in an independent manner, which imply structural semantics in the graph. Previous methods usually leverage a mean- or sum-pooling to aggregate these local representations, which could not capture the importance of local functional motifs in the protein graph. To tackle this, we adopt an attention module, which summarizes these semantics-aware super-node representations into graph representations in an adaptive fashion.

In detail, we utilize only one query vector $Q^{P}\in R^{1\times D}$. In formulation, we define two transformation matrices $K^{P}$ and $V^{P}\in R^{D\times D}$ and have:


(5) $$z=\text{softmax}\left(\frac{Q^{P}\cdot {\left(U\cdot K^{P}\right)}^{⊤}}{\sqrt{D}}\right)\cdot U\cdot V^{P},$$

In the last, we utilize an MLP to map each graph representation $z\in R^{D}$ to a predictive vector $\hat{y}\in R^{C}$ with a sigmoid activation function where *C* is the number of GO terms. Each element of $\hat{y}$ indicates the positive probability of each GO term.

### 2.5 Optimization with contrastive learning

In this part, we utilize graph contrastive learning to enhance the graph representation as a regularization and supervised loss is also involved.

**Graph Contrastive Learning.** Recently, graph contrastive learning has achieved superior results in unsupervised learning and pretraining for graph data (Zeng and Xie 2021, Yu *et al.* 2022). Inspired by this, we seek to utilize it for regularization in our model. To achieve this, we add random noise to the node vectors in the hidden embeddings which provide different views for each protein view without deleting significant residues and interactions (Yu *et al.* 2022). Then, we increase the similarity score between graph representations of two views compared with other graphs.

In detail, for each graph *G*, we inject noise to every node *v* in the graph to provide a different view. Formally, we first randomly sample the noise vector $\epsilon_{v}$ with $||\epsilon_{v}||=\epsilon$, and have: in which · denotes the element-wise product of two vectors. In Equation (6), we perturb node features in the same direction, which retains the key semantics in a smooth fashion. After perturbation, we can leverage $h_{v}^{\prime }$ to produce another view of the protein graph z′.


(6) $$h_{v}^{\prime }=h_{v}+|\epsilon_{v}|\cdot \mathrm{sign}(h_{v}),$$

Then we offer an objective to maximize the similarity between graph representations of different views compared with those of other graphs. In detail, we randomly select a minibatch of *M* graphs, each of which produces two views of graph representations. After reannotating ***z*** and z′ as $z_{m}$ and $z_{m}^{\prime }$ for the *m*-th protein in the minibatch, we adopt the InfoNCE loss function (He *et al.* 2020) for graph contrastive learning as follows: where *τ* denotes a temperature parameter set to 0.5 following (You *et al.* 2020) and ⋆ calculates the cosine similarity between two vectors. This term serves as regularization for discriminative graph representations, which has been proven to benefit downstream classification.


(7) $$L_{\text{reg}}=-\frac{1}{M}\sum_{m=1}^{M}\;\text{log}\;\frac{e^{z_{m}⋆z\prime_{m}/\tau }}{\sum_{m\prime =1}^{M}e^{z_{m}⋆z\prime_{m\prime }/\tau }},$$

**Supervised Loss.** Finally, we adopt a binary cross-entropy (BCE) loss objective for downstream multilabel classification as follows: where *y_ml_* and ${\hat{y}}_{ml}$ denote the ground truth and predicted positive probability for the *l*-th function of the *m*-th sample, respectively. The final loss function is derived by combining both supervised loss and regularization loss as:


(8) $$L_{\text{sup}}=-\frac{1}{M\cdot C}\sum_{l=1}^{C}\sum_{m=1}^{M}(y_{ml}\;\text{log}({\hat{y}}_{ml})+(1-y_{ml})\;\text{log}(1-{\hat{y}}_{ml})),$$

(9) $$L=L_{\text{sup}}+L_{\text{reg}}.$$

### 2.6 Model training

Overall, our models comprise of four graph convolutional layers, one hierarchical graph Transformer layer and one MLP module. We train the proposed HEAL using the Adam optimizer (Kingma and Ba 2014) with a learning rate of 0.0001 and a batch size of 64 for 100 epochs. All models are implemented by Pytorch and Pytorch geometric library (Fey and Lenssen 2019, Paszke *et al.* 2019). In order to prevent overfitting, we adopt an early-stopping criterion with patience of five epochs based on the validation set. All models are trained utilizing a single Tesla V100-SXM2 32GB graphics processing unit (GPU), with training times of approximately four hours using a batch size of 64.

## 3 Dataset

We first used the same dataset of DeepFRI (Gligorijević *et al.* 2021), which can be downloaded from https://github.com/flatironinstitute/DeepFRI. The dataset comprises of 36 641 protein structures from PDB database and 244 775 protein structures from SWISS-MODEL repository (Waterhouse *et al.* 2018), and we rename them as PDBch dataset and SMch dataset, respectively. All protein chains in the PDB database for which contact maps can be retrieved were downloaded, and the sequences were clustered at 95% sequence identity. Then, a representative PDB chain that has at least one functional annotation and high-resolution structure is selected into PDBch dataset. This dataset was partitioned into training, validation, and test sets at an 8:1:1 ratio. The experimentally solved structures of each sequence were fetched from PDB to construct the protein graph. The GO-term annotations were retrieved from SIFTS (Dana *et al.* 2019) and UniProtKB. A PDB model needs to share at least 90% sequence identity and cover at least 70% of the UniProtKB sequence to transfer the annotations. Each sequence was labeled with 489 Molecular Function (MF) terms, 1943 Biological Process (BP) terms, and 320 Cellular Component (CC) terms.

According to the frequency of each GO term appears in the PDBch training set (Supplementary Fig. S1), we computed information content (IC) of each GO term in the PDBch training set. The more specialized a GO term is, the higher IC it has.


(10) $$\text{IC}({\text{GO}}_{i})=-{\;\text{log}\;}_{2}(P({\text{GO}}_{i})).$$

The SMch dataset was constructed by obtaining homology models of the PDBch dataset with at least one annotation from the SWISS-MODEL repository. The similar SWISS-MODEL sequences were removed at 95% sequence identity. This dataset was partitioned into training and validation sets at a 9:1 ratio. According to the clustering result, the sequences in the PDBch test set can be divided into various homologous groups compared to the PDBch and SMch training set (sequence identity of 30%, 40%, 50%, 70%, and 95%).

We further tested whether recent development in protein structure prediction improves data augmentation. We selected 44 137 proteins with low-frequency GO terms (proteins with IC>10 in the PDBch training set) and retrieved their structures predicted by AlphaFold2 (AF2) from AlphaFold Protein Structure Database (Varadi *et al.* 2022). These protein chains constitute the AFch dataset. To partition the AFch dataset, we utilized MMseqs (Steinegger and Söding 2018) to cluster sequences at a sequence identity of 25%, resulting in an AFch training set with 43 072 sequences and an AFch test set with 567 sequences. We then removed any sequences in the AFch test set that had a sequence identity greater than 25% with any sequences in both the AFch training set and the PDBch training set. Finally, we randomly selected 10% of the sequences in the AFch training set to make up a validation set. The IC of each GO term in the combination of the AFch and PDBch training set can be found from Supplementary Fig. S2. More details about the datasets can be found from Supplementary Table S1.

## 4 Baseline methods

**Blast** (Altschul *et al.* 1990). We first remove all sequences similar to the test sequences from the training set using an E-value threshold of 1e-3. Next, we use the blastp program to identify the sequence with the highest score from the PDBch training set, and the predicted annotations of this sequence are scaled by the sequence identity to the query sequence to obtain the predicted annotations.

**FunFam** (Das *et al.* 2015). We search against CATH FunFams based on domain information. The annotations of the highest-scoring match are then transferred as the predicted result for the test sequence.

**DeepGO** (Kulmanov *et al.* 2018). DeepGO is a deep learning method that relies solely on the protein sequences. The sequences are represented as 1D sequential features, and 21 1D convolution layers are applied to predict protein functions. For our evaluation, the DeepGO model was trained on PDBch and SMch training sets from the DeepFRI study.

**DeepFRI** (Gligorijević *et al.* 2021). DeepFRI is a recently published GCN-based approach, which takes both sequences and structures as input to better capture spatial relations among residues. This method trains a protein language model to embed protein sequence, and a GCN model to learn the function prediction. For our evaluation, the DeepFRI model was trained on PDBch and SMch training sets from the DeepFRI study.

**DeepGOPlus** (Kulmanov and Hoehndorf 2021). DeepGOPlus is a hybrid method that combines the sequence homology-based method DIAMOND Blast (Buchfink *et al.* 2015) with a 1D convolutional neural network, similar to DeepGO. For our evaluation, we retrained DeepGOPlus using the PDBch and AFch training sets, and tuned the weight that combines the Diamond Blast score with the neural network score based on PDBch and AFch validation sets.

Considering that GAT-GO (Lai and Xu 2022) resplit the PDBch dataset and have not open source their code and dataset, so we cannot do the comparison with it.

## 5 Evaluation metrics

To evaluate these performance of different methods on PDBch test set, we use these main metrics: CAFA (Radivojac *et al.* 2013) evaluation metrics (i) protein-centric Fmax, (ii) Smin, and (iii) function-centric area under precision–recall (AUPR) curve. Protein-centric Fmax is the maximum F1 score over all prediction thresholds t∈[0,1] with a step size of 0.01. Smin represents the semantic distance between predicted and real annotations considering information content of each function. Function-centric AUPR is a reasonable measurement commmonly used for high-class-imbalance situation. Additional details on how to compute these metrics can be found in Supplementary Section 2.

## 6 Results

### 6.1 HEAL improves protein function prediction

We evaluate the performance of our model on the PDBch test set by comparing it with Blast, Fumfams, DeepGO, and DeepFRI. While DeepGO and DeepFRI were trained on both the PDBch and SMch training sets, our model (HEAL-PDB) is trained solely on the PDBch training set. We assess the performance of the models on three gene ontology domains (MF, BP, CC) separately. The results, as presented in Table 1, show that HEAL-PDB achieves Fmax scores of 0.691, 0.565, 0.655, Smin scores of 0.401, 0.540, 0.501, and AUPR scores of 0.571, 0.259, 0.342 on the MF, BP, and CC tasks, respectively. HEAL-PDB outperforms Blast, FunFams, and DeepGO across all three gene ontology domains. Compared to DeepFRI, HEAL-PDB performs significantly better on the MF and CC tasks, and shows comparable results on the BP task. Despite being trained on much less data, the architecture of our model demonstrates advantages in learning protein functions.

**Table 1.** AUPR, Fmax, and Smin of different methods on PDBch test set.^a^

Method	Training set	AUPR (↑)			Fmax (↑)			Smin (↓)		
		MF	BP	CC	MF	BP	CC	MF	BP	CC
Blast	–	0.136	0.067	0.097	0.328	0.336	0.448	0.632	0.651	0.628
FunFams	–	0.367	0.260	0.288	0.572	0.500	0.627	0.531	0.579	0.503
DeepGO	PDBch+SMch training set	0.391	0.182	0.263	0.577	0.493	0.594	0.472	0.577	0.550
DeepFRI	PDBch+SMch training set	0.495	0.261	0.274	0.625	0.540	0.613	0.437	0.543	0.527
HEAL-PDB	PDBch training set	0.571	0.259	0.342	0.691	0.565	0.655	0.401	0.540	0.501
HEAL-SW	PDBch+SMch training set	0.653	0.308	0.432	0.711	0.581	0.654	0.366	**0.509**	0.489
HEAL	PDBch+AFch training set	**0.691**	**0.337**	**0.467**	**0.747**	**0.595**	**0.687**	**0.342**	**0.509**	**0.458**

a Best performance in bold. Fmax and AUPR, highest; Smin, lowest.

When the SMch dataset was included for training, the resulting model HEAL-SW performs better than both HEAL-PDB and DeepFRI. As recently developed deep learning-based methods for protein structure prediction have become more reliable, we improved our model further by incorporating the AFch training set and the resulting model is referred as HEAL. HEAL achieves Fmax of 0.747, 0.595, 0.687, Smin of 0.342, 0.509, 0.458, and AUPR of 0.691, 0.337, 0.467 on MF, BP, CC tasks, which surpasses the state-of-the-art open-source GCN method DeepFRI by a significant margin, and even goes beyond HEAL-SW despite using much fewer proteins for training. These results indicate that HEAL leads the way in protein function prediction.

### 6.2 Ablation study

To investigate how different components of HEAL contribute to its performance, we conduct the ablation experiments on the PDBch test set. We introduce three variants as below: (i) HEAL w/o CL: it removes the contrastive learning objective. (ii) HEAL w MP: it utilizes the max pooling to replace HGT (iii) HEAL w/o EE: it removes ESM-1b embeddings from the node attributes. The results are summarized in Table 2. Removing contrastive learning module causes a moderate drop over all three gene ontology domains, because contrastive learning as a regularization trick, the noise it brings into GCN network can make HEAL more robust, and InfoNCE loss function can increase the variability of each graph embeddings. When we substitute the Hierarchical Graph Transformer module with the commonly used max pooling, the performance decreases significantly, which indicates our topological pooling manner is superior to the traditional node-equal-treatment pooling, and introduction of super-node representations is better at capturing key functional substructures. ESM-1b, as a widely applied protein language model, is capable of guiding protein engineering tasks, removing which leads to a drastic decrease at the efficiency of HEAL. AUPR and Fmax metrics of three tasks demonstrate that ESM-1b embeddings bring tremendous improvement to our model.

**Table 2.** Ablation study of HEAL on PDBch test set.^a^

Method	AUPR (↑)			Fmax (↑)			Smin (↓)		
	MF	BP	CC	MF	BP	CC	MF	BP	CC
HEAL	**0.691**	**0.337**	**0.467**	**0.747**	**0.595**	**0.687**	**0.342**	**0.509**	**0.458**
HEAL w/o CL	0.635	0.304	0.410	0.708	0.586	0.672	0.375	0.521	0.478
HEAL w/o MP	0.588	0.252	0.378	0.666	0.552	0.665	0.416	0.547	0.486
HEAL w/o EE	0.284	0.130	0.222	0.478	0.447	0.579	0.554	0.607	0.553

a Best performance in bold. Fmax and AUPR, highest; Smin, lowest. The three variants of HEAL are: (i) HEAL w/o CL (contrastive learning): it removes the contrastive learning objective. (ii) HEAL w MP (max pooling): it utilizes the max pooling to replace HGT (iii) HEAL w/o EE (ESM-1b embeddings): it removes ESM-1b embeddings from the node attributes.

### 6.3 Generalizability of HEAL

**Figure 2.**
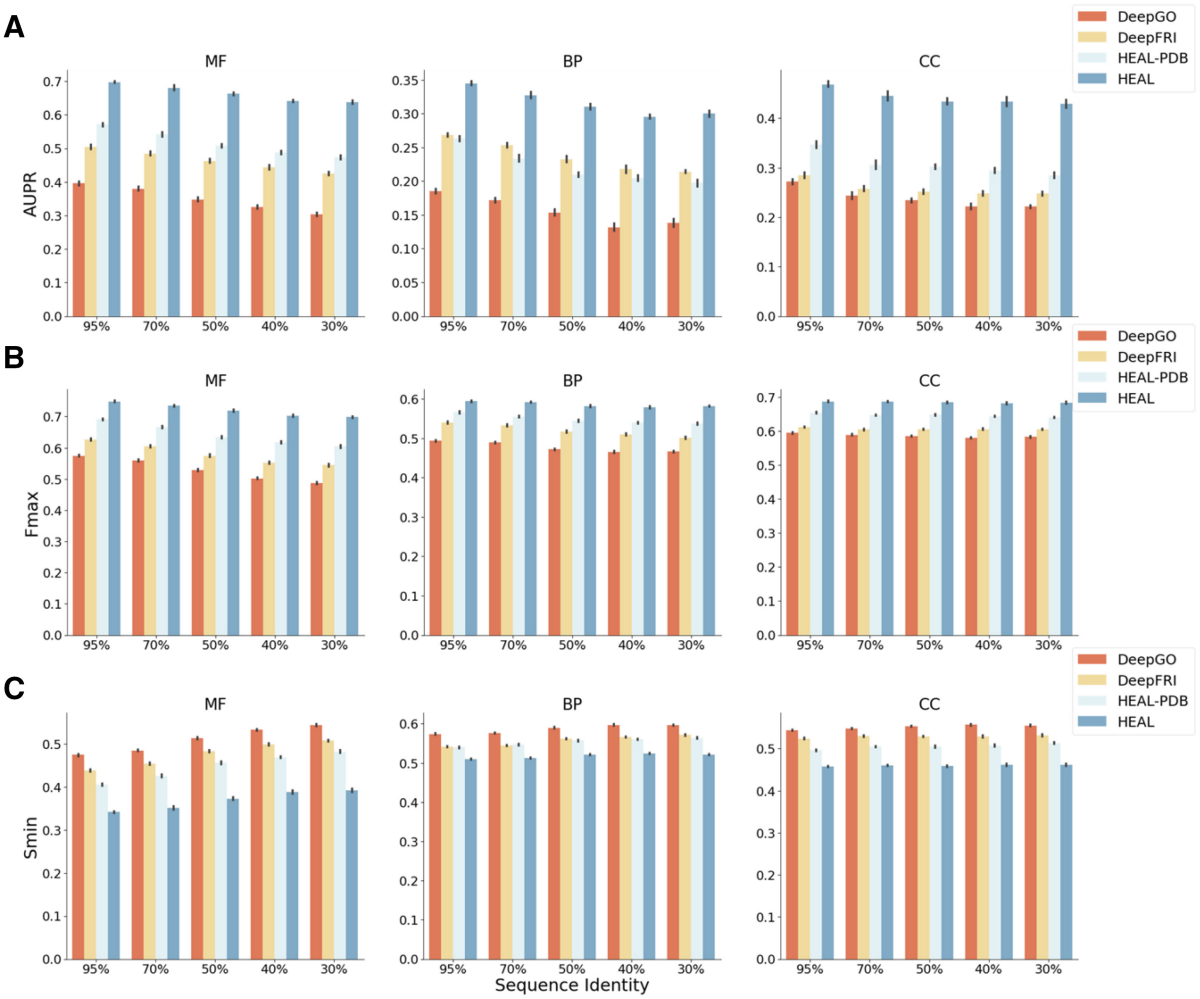
AUPR (A), Fmax (B), and Smin (C) of different methods on PDBch test set over different sequence identity thresholds.

In order to evaluate the generalizability of HEAL, we evaluate its performance on PDBch test sequences with varying homology to the combined PDBch and SMch training sets, which are the training set for DeepFRI and DeepGO models. The five sequence identity thresholds are 30%, 40%, 50%, 70%, and 95%. Given that both HEAL and HEAL-PDB were trained on subsets of the PDBch and SMch training sets, this comparison is valid. Notably, deep learning methods significantly outperform sequence alignment-based methods. Therefore, DeepFRI, DeepGO, and HEAL-PDB are also included in the comparison. Fmax, AUPR, and Smin are computed using 10 bootstrap iterations across all test proteins. As is depicted in Fig. 2A–C, compared to sequence-only based method DeepGO, HEAL-PDB and DeepFRI exhibit enhanced efficiency when incorporating spatial relationships between residues. HEAL-PDB outperforms DeepFRI and DeepGO over all homology thresholds at MF and CC task, while at BP task, DeepFRI surpasses HEAL-PDB over all five homology thresholds at BP task. With the augmentation of the AFch dataset, HEAL demonstrates the best performance across all five thresholds for the three gene ontology categories, even maintaining a significant lead over other methods (Supplementary Tables S3.1–S3.3). As homology decreases, the performance of HEAL declines more gradually, suggesting that the integration of a protein language model and additional high-quality structures facilitates HEALs ability to learn the relationship between structural and functional properties.

### 6.4 Performance of HEAL on GO terms with different specificity

We spilt proteins in the PDBch test set into three groups based on the IC of each term belonging to MF, BP, and CC tasks (IC>5, 5 < IC<10, and IC>10). Among all the three ranges, as depicted from Supplementary Fig. S3, HEAL-PDB outperforms DeepGO and achieves similar results with DeepFRI. Apparently, HEAL shows the best performance at countering specificity (Supplementary Table S3.4). On commonly occurring terms (IC<5), HEAL, HEAL-PDB, DeepFRI, and DeepGO have average AUPR of 0.790, 0.752, 0.732, and 0.673, respectively. On GO terms of medium IC (5 < IC<10), HEAL, HEAL-PDB, DeepFRI, and DeepGO have average AUPR of 0.506, 0.436, 0.404, and 0.313, respectively. Even on highly specific annotations as GO terms of IC>10, HEAL (0.321) performs significantly better than HEAL-PDB (0.214), DeepFRI (0.204), and DeepGO (0.137).

### 6.5 Performance of HEAL on AlphaFold2 predicted structures

A more realistic usage scenario for our methods is to predict biological functions for proteins with neither experimentally resolved structure nor annotated similar sequences. In this scenario, it is more appropriate to compare our methods with robust methods that rely on both sequence and homology information. To accomplish this, we retrained DeepGOPlus (Kulmanov and Hoehndorf 2021) on the same training set of HEAL. Then, we test the performance of our methods on AFch test set, as well as two other competing methods: DeepFRI and DeepGOPlus.

**Figure 3.**
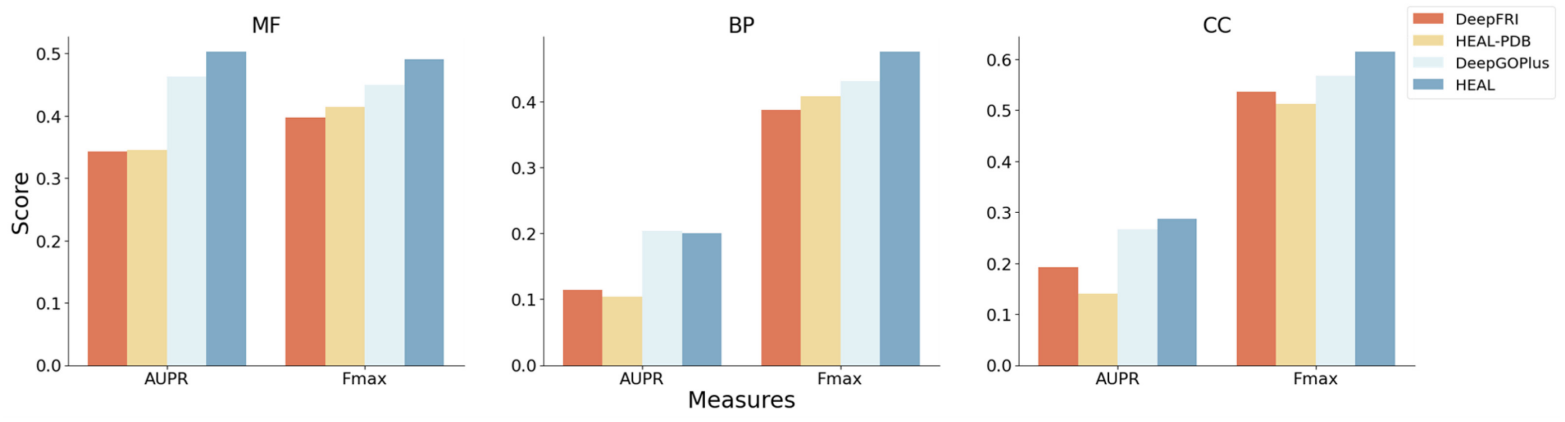
AUPR and Fmax of different methods on AFch test set.

As is shown from Fig. 3, although structures predicted by homology modeling of the sequences in AFch test set exist in the training set of DeepFRI, HEAL-PDB trained solely on PDB structures achieves similar performance with DeepFRI. Diamond Blast provides DeepGOPlus with a strong ability to transfer GO-term annotation from similar sequences. By combining the scores predicted by 1D CNN, DeepGOPlus outperforms both DeepFRI and HEAL-PDB. Our model HEAL not only gets obviously higher Fmax score (0.491, 0.475, 0.614) at all MF, BP, CC tasks than DeepGOPlus (0.450, 0.430, 0.567), but also achieves higher AUPR score, except that its AUPR score (0.200) for BP task is very slightly lower than DeepGOPlus (0.203) (Supplementary Table S3.5). The results indicate that HEAL can play a greater role in more realistic application scenarios.

### 6.6 Analysis of key residues in HEAL models

To localize the prediction result into each residue, we apply the gradient-weighted Class Activation Map (grad-CAM; Selvaraju *et al.* 2017). Grad-CAM was first proposed to provide visual explanations for CNN classifiers. It can highlight on which part of a picture the model makes the decision. In our scenario, grad-CAM can be used to find out which residues make more contributions for the concerned function. In grad-CAM, we choose the output of the last graph convolutional layer as the feature map, $F\in R^{L\times D}$, *L* denotes the length of the protein and *D* denotes the hidden dimension. Then we take the derivative of the protein function *y^l^* with respect to ***F*** as the gradient weight $W_{i,j}^{l}$:


(11) $$W_{i,j}^{l}=\frac{\partial y^{l}}{\partial F_{i,j}}$$

The contribution score of each residue $\mathrm{CA}M_{i}^{l}$ can be obtained by doing the weighted sum with $W_{i,j}^{{}^{l}}$ and $F_{i,j}$.


(12) $$\mathrm{CA}M_{i}^{l}=\mathrm{ReLU}(\frac{\sum_{j=1}^{D}W_{i,j}^{l}\cdot F_{i,j}}{D})$$

The function-specific heatmap will be normalized for each $\mathrm{CA}M_{i}^{l}$.

**Figure 4.**
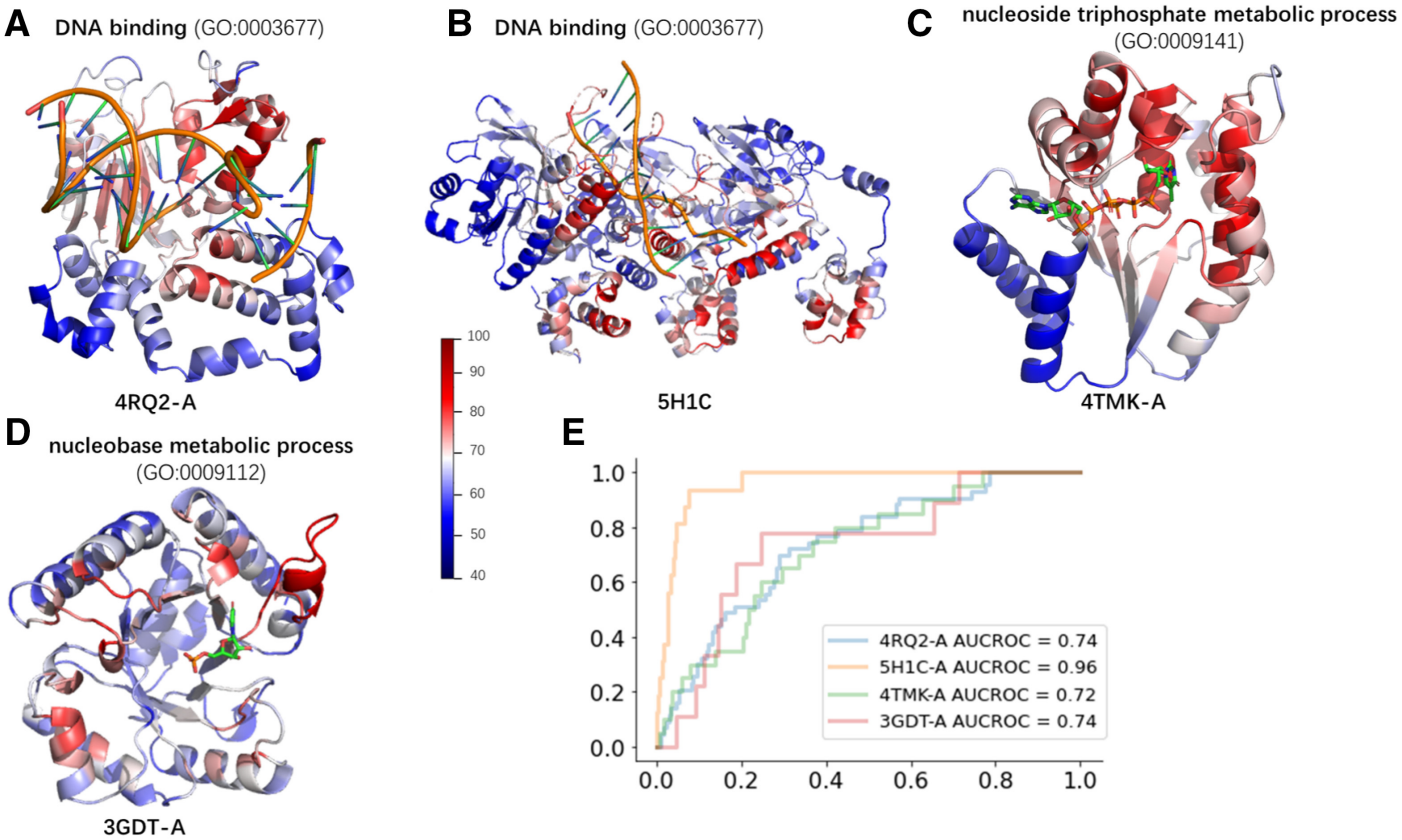
Four examples of the grad-CAM heat map mapped onto the experimentally solved structures. All the residues are colored according to contribution score computed through grad-CAM. More salient residues are emphasized, while less salient residues are de-emphasized. (A) and (B) Examples of DNA-binding proteins (PDB Id: 4RQ2, 5H1C). (C) An example of the protein engaged in nucleoside triphosphate metabolic process (PDB Id: 4TMK), the small molecule is a triphosphate analog. (D) An example of the protein engaged in nucleobase metabolic process (PDB Id: 3GDT), the small molecule is UP6. (E) ROC curves indicate that contribution scores computed by grad-CAM overlap with binding sites retrieved from the BioLiP database.

For MF-GO terms, we provide two cases where their heatmaps are consistent with the experimentally confirmed binding sites. The first example is 4RQ2, a single-chain DNA polymerase with the function of DNA-binding (GO:0003677). As is shown in Fig. 4A, we projected the heatmap onto the protein structure and observed strong signals in regions where DNA binds. The second example is 5H1C, a DNA repair protein RAD51 homolog that functions as a homotrimer. “High-temperature” regions also concentrated surrounding the DNA in spite of some strong signals away from DNA (Fig. 4B).

For BP-GO terms, we offer two examples. The first example is 4TMK, a thymidylate kinase involved in the nucleoside triphosphate metabolic process (GO:0009141). As shown in Fig. 4C, the residues around the inhibitor TP5A contribute significantly to the heatmap. The second example is 3GDT, a phosphate decarboxylase that plays an important role in nucleobase metabolic process (GO:0009112). Its UP6-binding site corresponds to the heatmap signal (Fig. 4D).

We extracted the binding sites of the four proteins from the BioLiP database (Yang *et al.* 2013). We then scale the contribution score from grad-CAM into 0–100 (Supplementary Fig. S4), and use receiver operating characteristic (ROC) curves to compare the high-contribution residues by grad-CAM to those involved in the experimentally verified binding sites. As is shown in Fig. 4E, area under the ROC curve (AUC-ROC) illustrates that our model has excellent capability to capture binding residues.

As the HEAL models were trained on both experimentally solved and AF2 predicted structures, it can also predict the key binding sites from AF2 predicted protein structures. A0A3P7DWR6 is another DNA repair protein RAD51 homolog, and its structures are unavailable from PDB. Our model can recall functions of A0A3P7DWR6 confidently with its AF2 predicted structures as input. The conservativeness of DNA binding sites between A0A3P7DWR6 and 5H1C suggests that they share the same binding mode (Supplementary Fig. S5), and the grad-CAM heatmap can still identify the core binding sites (Supplementary Fig. S6).

## 7 Discussion

In this study, we have proposed the contrastive learning assisting GCN model HEAL, and introduced the hierarchical graph Transformer to conduct node aggregation and graph pooling. By integrating protein structure and sequence language embeddings, HEAL provides a powerful tool for protein function prediction, which significantly outperformed the state-of-art model DeepFRI. In addition, HEAL demonstrates better generalizability to sequences that are dissimilar from those in the training set, as well as superior specificity-resistance for infrequent function prediction. Owing to the close relationship between structures and functions, addressing the issue from a structural perspective circumvents the complex challenge of discerning long-term correlations in sequence data. Furthermore, protein language models inherently acquire evolutionary information through self-supervised learning. This intrinsic characteristic significantly enhances the capacity of neural network models to effectively capture and comprehend the evolutionary patterns underlying functional motifs (Lai and Xu 2022, Zhu *et al.* 2022, Wang *et al.* 2023). When integrated with an extensive collection of high-quality protein structures, as predicted by AlphaFold2, our model acquires a more comprehensive understanding of structural patterns corresponding to their functions. Consequently, our model HEAL demonstrates enhanced generalization capabilities.

On the AFch test set, which includes AF2 predicted structures with low sequence similarity to the training set and no experimentally resolved structures, HEAL exhibits remarkable robustness and outperforms other state-of-the-art methods. This result suggests that HEAL has great potential for application in real-world scenarios.

By introducing the grad-CAM method, we find that our model can identify functional residues that correspond well with experimentally confirmed residues. When the AF2 predicted protein structures are utilized, our model still exhibits good interpretability.

To infer protein functions using HEAL, either experimentally solved structures or AF2 predicted structures are required as input, which adds an additional step compared to sequence-based methods. However, the recent breakthrough in protein structure prediction using large protein language models (Lin *et al.* 2023) suggests that it may be possible to accurately predict protein functions based solely on primary sequences. Additionally, there are a vast number of annotated sequences whose structures have yet to be resolved, which could provide ample training data to further enhance the prediction models. In the future, we aim to modify single sequence structure prediction models so that the learned evolutionary and structural information can be leveraged to annotate more sequences in larger datasets such as CAFA.

## Supplementary Material

btad410_Supplementary_Data
