# Supplementary material for: Hierarchical graph transformer with contrastive learning for protein function prediction
Source: Bioinformatics. 2023 Jun 27;39(7):btad410. doi: 10.1093/bioinformatics/btad410 (PMC10338137; doi:10.1093/bioinformatics/btad410)
Supplement: btad410_Supplementary_Data [file btad410_supplementary_data.pdf]

Supplementary Information for

Hierarchical Graph Transformer with Contrastive Learning for Protein Function Prediction

Zhonghui Gu[1, †], Xiao Luo[2, †], Jiaxiao Chen[3], Minghua Deng[3,4, *], Luhua Lai[1,3,5, *]

[1]Peking-Tsinghua Center for Life Sciences, Academy for Advanced Interdisciplinary Studies, Peking University, Beijing, 100871, China
[2]Department of Computer Science, University of California, Los Angeles, 90024, USA
[3]Center for Quantitative Biology, Academy for Advanced Interdisciplinary Studies, Peking University, Beijing, 100871, China
[4]School of Mathematics Sciences, Peking University, Beijing, 100871, China
[5]BNLMS, College of Chemistry and Molecular Engineering, Peking University, Beijing, 100871, China

[†]Equal Contribution.

[*]Corresponding authors: lhlai@pku.edu.cn or dengmh@math.pku.edu.cn

This file contains the following information:

1. The composition of the datasets.
2. Details of evaluation metrics.
3. Information content distribution in the training set.
4. Detailed data for performance comparison.
5. Plots for contribution score computed by grad-CAM method.
6. Grad-CAM score mapped onto AlphaFold2 (AF2) predicted structures.

1. Dataset

Table S1. Numbers of sequences in the datasets

Datasets	Number of sequences		
	Training	Testing	Validation
PDBch	29,893	3,414	3,322
SMch	220,297	-	24,478
AFch	38, 185	567	4,242

The number of sequences in PDBch dataset is a little different from DeepFRI article, because the structure of 5JM5 was removed from PDB database, and there exists some problems like unknown residues for whole chains in the structure of 6BBM, 6GML and 6GMH.

2. Evaluation metrics

2.1 F_{max}

$$F_{\max} = \max_{t} \left\{ \frac{2 \cdot AvgPr(t) \cdot AvgRc(t)}{AvgPr(t) + AvgRc(t)} \right\} \quad (1)$$

$$AvgPr(t) = \frac{1}{m(t)} \cdot \sum_{i=1}^{m(t)} pr_i(t) \quad (2)$$

$$AvgRc(t) = \frac{1}{n} \cdot \sum_{i=1}^{n} rc_i(t) \quad (3)$$

F_{max} is the max F1-score over all thresholds (eq(1)). The average precision score $AvgPr(t)$ and average recall score $AvgRc(t)$ at the threshold t follow eq(2) and eq(3) respectively, where $pr_i(t)$ and $rc_i(t)$ represent precision score and recall score for protein i respectively. t ranges from 0 to 1 with a step size of 0.01. n is the number of proteins in the test set, and $m(t)$ is the number of proteins predicted with at least one function.

2.2 $AUPR$

We utilize the macro-AUPR to measure the performance. macro-AUPR is the area under the $AvgPr'(t)$ and $AvgRc'(t)$ curve. Different from $AvgPr(t)$ and $AvgRc(t)$ which are averaged over each protein, $AvgPr'(t)$ and $AvgRc'(t)$ are the precision score and recall score at the threshold t averaged over each function.

2.3 S_{min}

S_{min} computes the semantic distance between real and predicted annotations according to information content (IC) of each class (eq(7)), which follows eq(4). In eq(4), $ru(t)$ represents the average remaining uncertainty (eq(5)) and $mi(t)$ represents average misinformation (eq(6)). T_i is the true annotations for the protein i, $P_i(t)$ is the predicted annotations for the protein i.

$$S_{\min} = \min_t \sqrt{ru(t)^2 + mi(t)^2} \quad (4)$$

$$ru(t) = \frac{1}{n}\sum_{i=1}^{n} \sum_{c \in T_i - P_i(t)} IC(l) \quad (5)$$

$$mi(t) = \frac{1}{n}\sum_{i=1}^{n} \sum_{c \in P_i(t) - T_i} IC(l) \quad (6)$$

$$IC(l) = -log_2(P(l)) \quad (7)$$

3. Distribution of IC in the training set

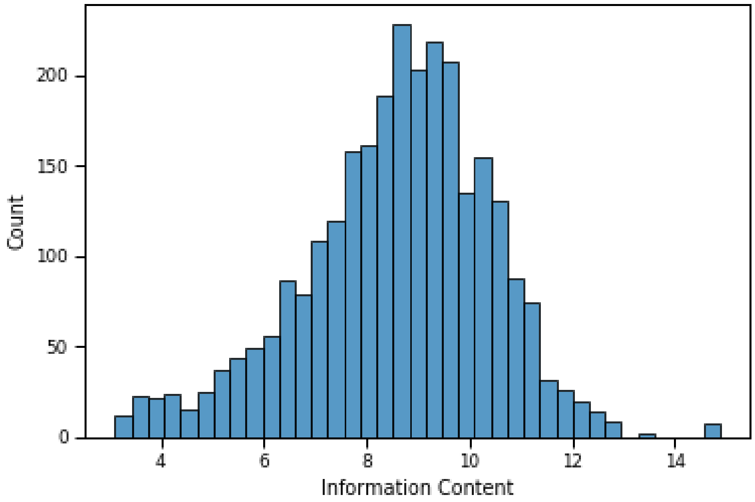

Fig. S1. Distribution of Information Content (IC) for protein functions in the PDBch training set.

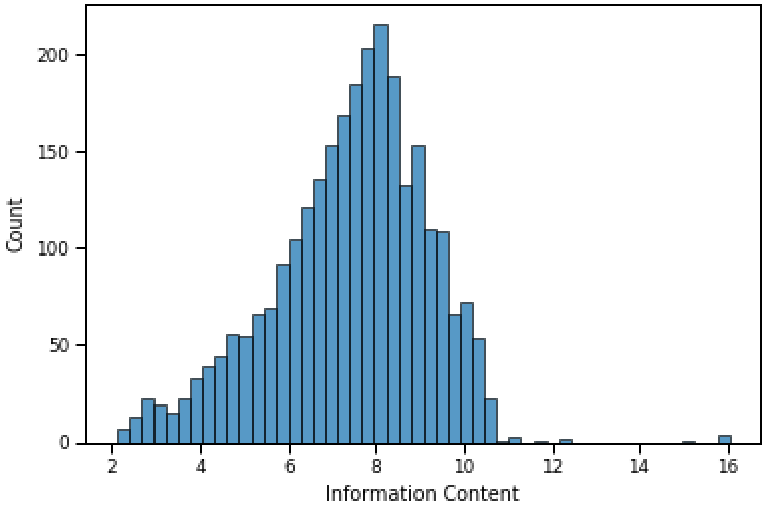

Fig. S2. Distribution of Information Content (IC) for protein functions in the combination of PDBch training set and AFch training set.

4. Data for performance comparison

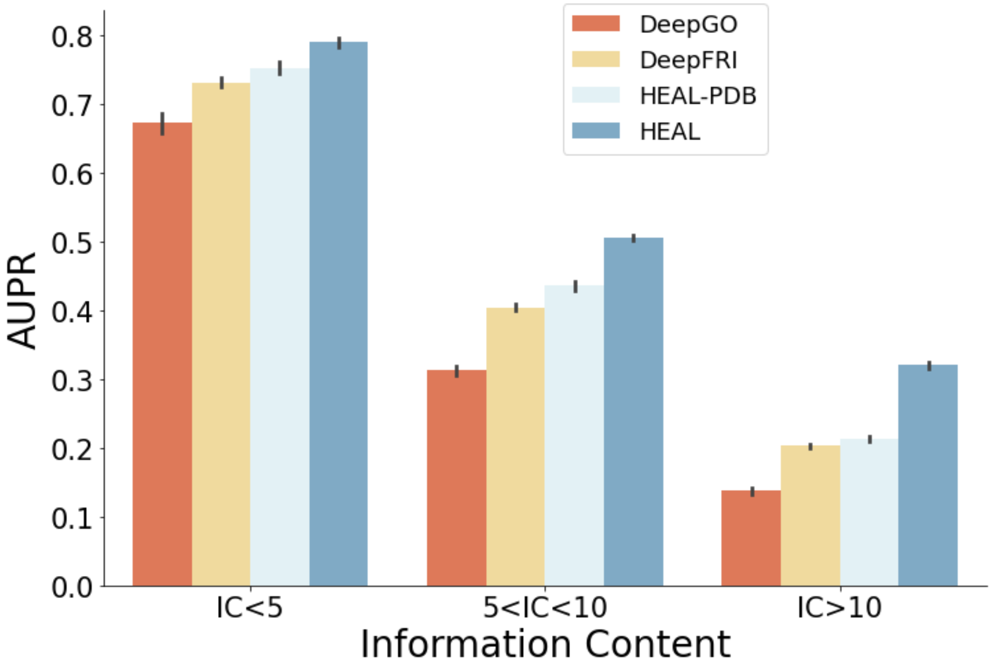

Fig. S3. AUPR of different methods on PDBch test set over different IC (information content) thresholds.

Table S3.1 Average macro-AUPR of HEAL and other competing methods on PDBch test set under five homology threshold to the training set for 10-bootstrap sampling

			AUPR of MF ontology		
Model	<30%	<40%	<50%	<70%	<95%
DeepGO	0.303	0.326	0.347	0.380	0.395
DeepFRI	0.425	0.443	0.463	0.485	0.504
HEAL-PDB	0.474	0.487	0.507	0.541	0.571
HEAL	0.638	0.641	0.663	0.681	0.698
			AUPR of BP ontology		
	<30%	<40%	<50%	<70%	<95%
DeepGO	0.138	0.132	0.154	0.171	0.185
DeepFRI	0.214	0.218	0.232	0.253	0.268
HEAL-PDB	0.197	0.205	0.209	0.233	0.263
HEAL	0.300	0.296	0.311	0.327	0.345
			AUPR of CC ontology		
	<30%	<40%	<50%	<70%	<95%
DeepGO	0.221	0.222	0.234	0.244	0.272
DeepFRI	0.248	0.248	0.251	0.258	0.285
HEAL-PDB	0.285	0.294	0.302	0.306	0.347
HEAL	0.429	0.434	0.434	0.445	0.468

Table S3.2 Average Fmax of HEAL and other competing methods on PDBch test set under five homology threshold to the training set for 10-bootstrap sampling

Fmax of MF ontology					
Model	<30%	<40%	<50%	<70%	<95%
DeepGO	0.487	0.501	0.528	0.559	0.575
DeepFRI	0.544	0.552	0.575	0.604	0.626
HEAL-PDB	0.604	0.617	0.634	0.667	0.691
HEAL	0.698	0.702	0.719	0.735	0.749
Fmax of BP ontology					
	<30%	<40%	<50%	<70%	<95%
DeepGO	0.466	0.466	0.472	0.489	0.494
DeepFRI	0.502	0510	0.517	0.533	0.540
HEAL-PDB	0.537	0.539	0.544	0.555	0.566
HEAL	0.582	0.578	0.582	0.592	0.594
Fmax of CC ontology					
	<30%	<40%	<50%	<70%	<95%
DeepGO	0.583	0.581	0.586	0.589	0.595
DeepFRI	0.605	0.606	0.606	0.605	0.612
HEAL-PDB	0.640	0.644	0.648	0.647	0.654
HEAL	0.684	0.682	0.684	0.686	0.687

Table S3.3 Average Smin of HEAL and other competing methods on PDBch test set under five homology threshold to the training set for 10-bootstrap sampling

			Smin of MF ontology		
Model	<30%	<40%	<50%	<70%	<95%
DeepGO	0.543	0.532	0.512	0.484	0.474
DeepFRI	0.507	0.498	0.483	0.454	0.438
HEAL-PDB	0.481	0.469	0.456	0.425	0.405
HEAL	0.391	0.388	0.372	0.351	0.341
			Smin of BP ontology		
	<30%	<40%	<50%	<70%	<95%
DeepGO	0.597	0.596	0.590	0.576	0.574
DeepFRI	0.571	0.566	0.561	0.545	0.541
HEAL-PDB	0.564	0.560	0.557	0.547	0.540
HEAL	0.522	0.524	0.521	0.512	0.510
			Smin of CC ontology		
	<30%	<40%	<50%	<70%	<95%
DeepGO	0.555	0.557	0.553	0.548	0.544
DeepFRI	0.531	0.529	0.528	0.530	0.524
HEAL-PDB	0.513	0.508	0.505	0.505	0.496
HEAL	0.461	0.461	0.458	0.461	0.458

Table S3.4 macro-AUPR of HEAL and other competing methods on PDBch test set under different IC ranges

		AUPR	
	IC<5	5<IC<10	IC>10
DeepGO	0.673	0.313	0.137
DeepFRI	0.732	0.404	0.204
HEAL-PDB	0.752	0.436	0.214
HEAL	0.790	0.506	0.321

Table S3.5 macro-AUPR and Fmax of HEAL and other competing methods on AFch test set

	MF		BP		CC	
	AUPR	Fmax	AUPR	Fmax	AUPR	Fmax
DeepFRI	0.342	0.398	0.114	0.387	0.192	0.536
HEAL-PDB	0.345	0.414	0.104	0.408	0.141	0.513
DeepGOPlus	0.463	0.450	0.203	0.430	0.267	0.567
HEAL	0.502	0.491	0.200	0.475	0.287	0.614

5. Plots for interpretability of HEAL

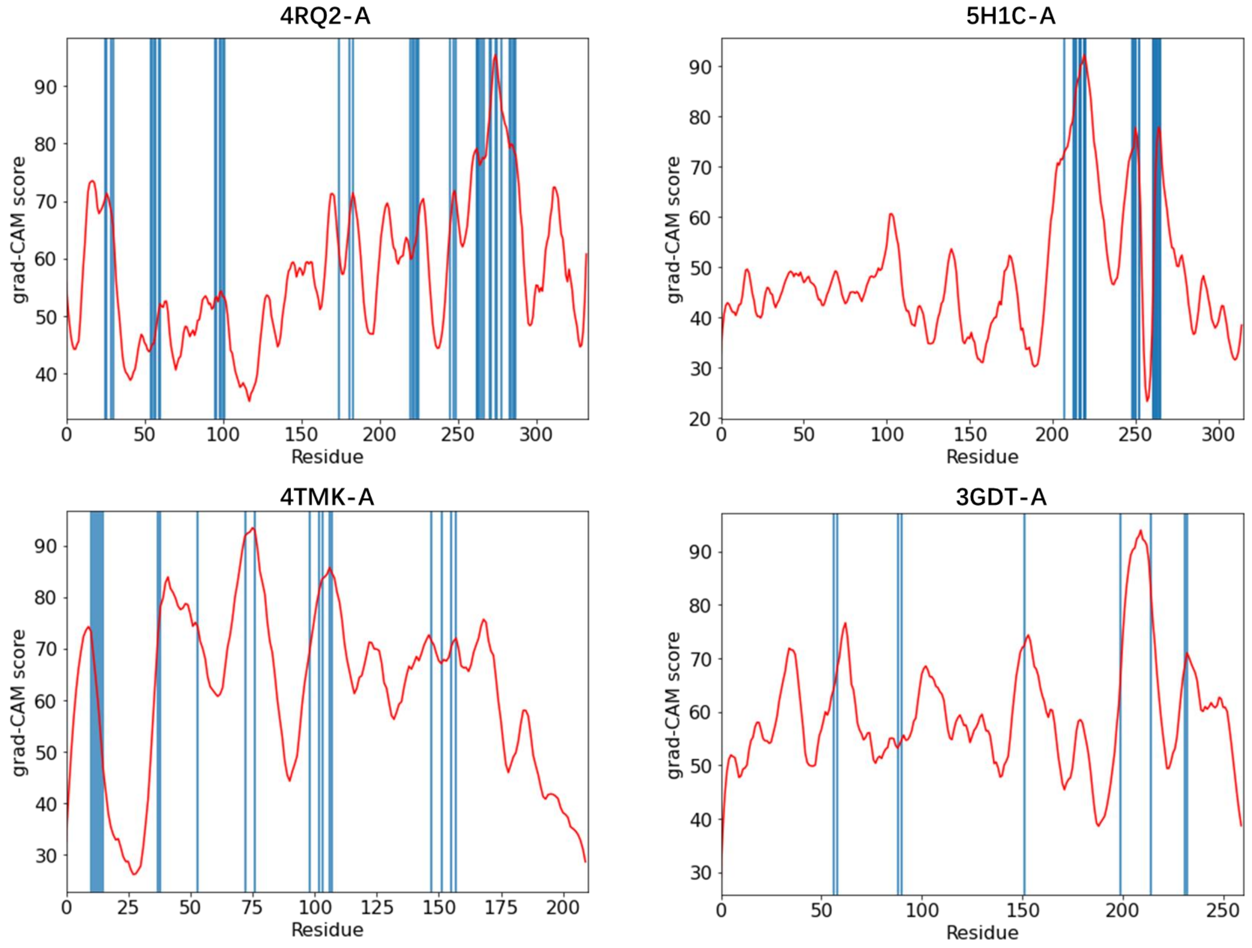

Fig. S4. Contribution score computed by grad-CAM for four protiens with expermentally-solved structures. The red line is the score computed by grad-CAM, and the blue line is the binding sites stored by the BioLiP database.

6. Grad-CAM score mapped onto AF2 predicted structures

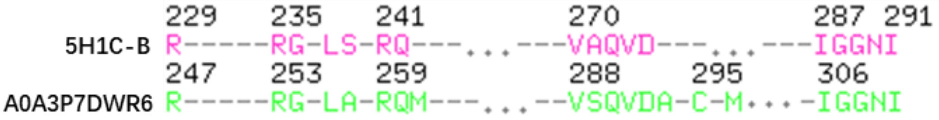

Fig. S5. The conservativeness of binding sites between A0A3P7DWR6 and 5H1C. The sequence identity between A0A3P7DWR6 and 5H1C is 55%. We analyze the sequence similarity between real binding sites and corresponding sites of AF2 predicted homologous structures through aligning experimentally solved complex structures with AF2 predicted homologous structures. We find that the binding sites are very conservative, which means it's highly possible that they share the same binding mode.

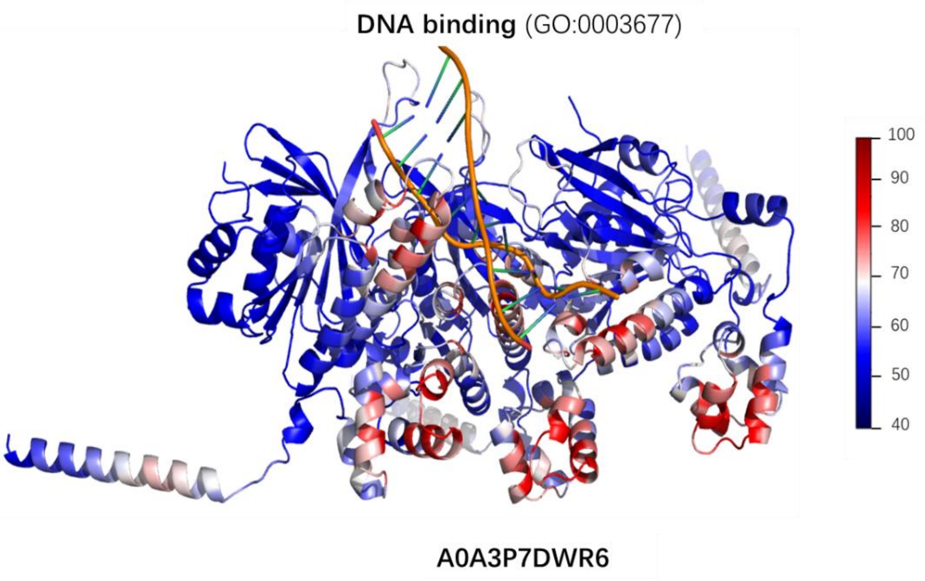

Fig. S6. The example of the grad-CAM heat map mapped onto the structure predicted by AF2. An examples of DNA-binding proteins homologous to 5H1C (Uniprot accession: A0A3P7DWR6). The coordinates of the DNA are determined by aligning the structures predicted by AF2 to its homologous complexes.
